# Mr. Henry Earle on the Re-Establishment of a Canal in the Urethra

**Published:** 1822-03-01

**Authors:** 


*On the Re-establishment of a Canal in the place of a portion of the Urethra which had been destroyed.* By Henry Earle, Esq. Surgeon to the Foundling Hospital, and Assistant Surgeon to St. Bartholomew's Hospital. Quarto, pp. 13. From the Philosophical Transactions, 1821.

The male urethra, as Mr. Earle justly observes, being a part which is called into action after very short intervals of repose, any impediment to its functions must be productive of constant sufferings. Affections too of the urinary organs, exert a more than commonly depressing influence on the human mind, since these organs are destined to perform a double office in the animal economy, one of which, is no less than the propagation of the species. In the following case the patient was raised from a state of great despondency to comparative happiness.

*Case.* John Whittaker, a seaman, fell with one leg on each side of the gunwale of a boat, in May 1813, by which accident the urethra was so injured in the perineum, that he was forced to have the catheter introduced for six weeks. From that time he continued to experience more or less difficulty in discharging his urine; and in May 1819, he was attacked with a sudden ischuria, which was soon followed by extensive effusion into the cellular substance. Mortification took place, and the integuments in the perineum, with about an inch of the canal of the urethra, sloughed away. A free vent being thus obtained, the mischief did not extend itself to the scrotum. Several unsuccessful attempts, by the gentleman in attendance, were made during the healing process, to unite the integuments over the catheter. In the month of August following, the patient came under the care of our author in St. Bartholomew's hospital, with a large smooth cicatrix occupying the urethra, no vestige of which remained at that part. The mucous membrane of the canal was distinctly visible, terminating above, and recommencing below the cicatrix. Through the posterior aperture the whole of the urinal and seminal discharges were evacuated, the canal anterior to the breach being increased in density, contracted, and likely soon to be obliterated. Our author determined on pursuing the following plan. The integuments on the right side had suffered less than those on the left, so that when a catheter was introduced, that portion which passed across the cicatrix could be about half covered by drawing the skin and cicatrix from the right towards the opposite side. Our author's first attempt was to encourage this disposition in the integuments to fold over; and, as some delay was required to dilate the anterior part of the urethra with bougies, he was directed to remain in bed with his knees tied together over a pillow, and a truss was so applied as constantly to press the integuments from the right to the left side.

"After some weeks, the urethra being sufficiently dilated to admit a moderate sized catheter, I determined to attempt the following operation. The smooth cicatrized surface having become insensible to the irritation of the urine, I resolved to employ it in the formation of a canal, and to endeavour to connect by it the two portions of the urethra: for, as many months had elapsed since the healing of the wound, all contraction in the cicatrix had ceased, and it was probable that a passage formed of such parts would not be liable to any farther diminution in its calibre.

"On the other hand, I had to contend with two great difficulties: in the first place, the portion of cicatrized integument intended to be separated, was not of original formation; consequently, it was endued with less vital energy, and possessed fewer blood-vessels:

secondly, it was not possible to allow the parts to be at rest for the completion of any curative process for many hours together: the force also with which the urine was expelled, and the acrid nature of that discharge, were alike unfavourable to the cure by adhesive inflammation. All these circumstances having been well considered, a portion of integument was removed about an inch and half long, and one-third of an inch in width, on the left side of the cicatrix; the groove thus formed being intended to receive the edge of skin to be detached from the opposite side. An incision was then made across the perineum above and below, so as to pare away the callous edges of the urethra. The cutis was next dissected off from a portion of integument on the right side of the perineum, about an inch and half in length and half an inch broad, leaving a smooth space of rather more than an inch between the cut surfaces, which was intended to form the lining of the new canal. The integuments on the right side were now dissected up, turned over a catheter, and brought in contact with the opposite groove. The detached portion of cicatrix bled little during the operation, and, before it could be applied to the groove, the edge had so livid an appearance as to create an apprehension that it must perish. Two ligatures were employed to assist in retaining it in the desired position, and some straps of adhesive plaster and a bandage completed the dressings. The day following the operation, it was evident that some urine had escaped by the side of the catheter; and on the third day, when it became necessary to remove the dressings, it was found that the portion of the flesh which had been denuded of skin had sloughed, but that a sufficient quantity had united above and below to form a canal open at one side, and large enough to include the whole catheter." 7.

This was as favourable a result as could be expected under existing circumstances. The two surfaces, from whence the integuments had been removed, were now suffered to heal, but as the cicatrix on the right contracted, it drew the newly formed canal rather to that side, and tended to increase the opening into it. Soon after this the patient became much disordered in his health, and had an attack of lepra vulgaris, on which account nothing was attempted for some months, except several times freely excoriating the edges of the canal, and thus endeavouring to unite them by keeping them in contact. In this Mr. Earle was constantly foiled by the astonishing rapidity with which the skinning process took place from within outwards—apparently arising from the moist state in which the parts were constantly kept.*

---

" * In corroboration of this," says Mr. Earle, "I have lately employed bread and water poultices to healthy sores, which have skinned over with greater rapidity than under any other application. Since making these experiments, I have learnt that Professor Kern, of Vienna,

In the summer of 1820 another operation was deemed adviseable.

"A deep groove was made on the right side, the surface was denuded of its cutis to some extent, a considerable portion of integument was then detached from the left side, and, in order to obtain healthy skin, I encroached a little on the thigh, and laid bare the edge of the fascia lata. Instead of passing any ligature through the detached portion, the old quill suture was employed, which was passed from the two outer cut surfaces. A pad of adhesive plaster was interposed between the ligatures and the flap of skin, to diffuse the pressure more generally; and my patient, being now quite an adept in passing the catheter, was directed to introduce it about three times in the twenty-four hours, instead of retaining it in the bladder, which had permitted some of the urine to pass insensibly away, and had acted prejudicially in the former operation. By this attempt much more was gained, and about two-thirds of the canal were completed; still, however, there remained a small aperture at the upper part. We again attempted to close this by denuding the edges with escharotics and the lancet, but it skinned over too rapidly to allow of any union between the opposite surfaces. A third operation on a smaller scale was therefore necessary, which so nearly completed the cure as to leave only an orifice large enough to admit a bristle, which has subsequently closed, and, at the present time, (March 1821,) he remains perfectly well, and is able to expel the contents of his bladder *pleno rivo*." 9.

The above operation is very creditable to the ingenuity and perseverance of Mr. Earle. The breach in the urethra of Mr. Earle's patient appears to have been larger than in the cases operated on by Sir Astley Cooper, and published in that gentleman's Surgical Essays.

---

employs no other local remedy in the cure of ulcers, than water and a simple covering of linen. It is a curious fact, that in the sixteenth century, when the art of surgery was encumbered with useless nostrums and complicated instruments, and when the actual cautery and hot oils were the favourite remedies, that a similar simplicity of treatment should have been employed by Maistre Doublet, a contemporary of Ambrose Parey, of whom Brantome tells us.

"'Et toutes ses cures faisoit le dit Goublet par un simple linge blanc et belle eau simple de la fontaine ou des puits.'"

